# A prediction model for 5-year cardiac mortality in patients with chronic heart failure using ^123^I-metaiodobenzylguanidine imaging

**DOI:** 10.1007/s00259-014-2759-x

**Published:** 2014-03-25

**Authors:** Kenichi Nakajima, Tomoaki Nakata, Takahisa Yamada, Shohei Yamashina, Mitsuru Momose, Shu Kasama, Toshiki Matsui, Shinro Matsuo, Mark I. Travin, Arnold F. Jacobson

**Affiliations:** 1 Department of Nuclear Medicine, Kanazawa University Hospital, 13-1 Takara-machi, Kanazawa, 920-8641 Japan; 2 Second Department of Internal Medicine (Cardiology), Sapporo Medical University School of Medicine, Sapporo, Japan; 3 Department of Cardiology, Osaka Prefectural General Medical Center, Osaka, Japan; 4 Department of Cardiovascular Medicine, Toho University Omori Medical Center, Tokyo, Japan; 5 Department of Nuclear Medicine, Tokyo Women’s Medical University, Tokyo, Japan; 6 Department of Cardiology, Cardiovascular Hospital of Central Japan, Shibukawa, Japan; 7 Department of Cardiology, Social Insurance Shiga General Hospital, Otsu, Japan; 8 Department of Cardiology and Nuclear Medicine, Montefiore Medical Center, Albert Einstein Medical College, Bronx, NY USA; 9 Medical Diagnostics, GE Healthcare, Princeton, NJ USA; 10 Present Address: Department of Cardiology, Hakodate-Goryoukaku Hospital, Hakodate, Japan

**Keywords:** Chronic heart failure, ^123^I-Metaiodobenzylguanidine (MIBG), Cardiac mortality, Prediction model, Risk stratification

## Abstract

**Purpose:**

Prediction of mortality risk is important in the management of chronic heart failure (CHF). The aim of this study was to create a prediction model for 5-year cardiac death including assessment of cardiac sympathetic innervation using data from a multicenter cohort study in Japan.

**Methods:**

The original pooled database consisted of cohort studies from six sites in Japan. A total of 933 CHF patients who underwent ^123^I-metaiodobenzylguanidine (MIBG) imaging and whose 5-year outcomes were known were selected from this database. The late MIBG heart-to-mediastinum ratio (HMR) was used for quantification of cardiac uptake. Cox proportional hazard and logistic regression analyses were used to select appropriate variables for predicting 5-year cardiac mortality. The formula for predicting 5-year mortality was created using a logistic regression model.

**Results:**

During the 5-year follow-up, 205 patients (22 %) died of a cardiac event including heart failure death, sudden cardiac death and fatal acute myocardial infarction (64 %, 30 % and 6 %, respectively). Multivariate logistic analysis selected four parameters, including New York Heart Association (NYHA) functional class, age, gender and left ventricular ejection fraction, without HMR (model 1) and five parameters with the addition of HMR (model 2). The net reclassification improvement analysis for all subjects was 13.8 % (*p* < 0.0001) by including HMR and its inclusion was most effective in the downward reclassification of low-risk patients. Nomograms for predicting 5-year cardiac mortality were created from the five-parameter regression model.

**Conclusion:**

Cardiac MIBG imaging had a significant additive value for predicting cardiac mortality. The prediction formula and nomograms can be used for risk stratifying in patients with CHF.

**Electronic supplementary material:**

The online version of this article (doi:10.1007/s00259-014-2759-x) contains supplementary material, which is available to authorized users.

## Introduction

Chronic heart failure (CHF) remains a major cause of death in current medical practice. Improving survival while maintaining good quality of life is an important goal for the medical community. With respect to diagnostic tools for assessing cardiac status, a number of clinical examinations and biochemical tests can provide information on the functional status of patients with CHF [1]. In addition to effective drug interventions using beta-blockers and/or renin-angiotensin-aldosterone inhibitors, nonpharmacological interventions have been shown to significantly reduce cardiac mortality [2]. However, due to the high cost of many such interventions, particularly the use of implanted devices, these recent advances have increased the importance of appropriate identification of patients at high-risk of lethal events.

Among the various cardiac imaging modalities, ^123^I-metaiodobenzylguanidine (MIBG) imaging has the unique feature of visualizing sympathetic nervous function. MIBG shares pathways with noradrenaline for uptake, storage and release in sympathetic nerve endings [3]. Hyperactivity of sympathetic nerve function results in decreased cardiac MIBG uptake, a finding associated with an increased occurrence of unfavorable cardiac events including pump failure and lethal cardiac arrhythmias [4–6]. Both prospective studies and meta-analyses have shown that MIBG imaging results can be used to predict fatal cardiac events and serious arrhythmias [7–9].

Despite its documented prognostic power [10], MIBG imaging is under-utilized in clinical practice, primarily because the benefit of this procedure in addition to conventional clinical parameters including heart failure symptoms, left ventricular function and biochemical data is not clearly defined. However, several recent analyses have shown that MIBG results provide additional prognostic value to the validated multivariable Seattle Heart Failure model [11, 12]. We have compiled a pooled MIBG cohort database based on studies performed at six Japanese institutions with follow-up of up to 15 years [13]. Our analyses confirmed that MIBG is a potent predictor of all-cause death.

This study focused on cardiac death and in particular the prediction of fatal cardiac events by adding information from MIBG imaging. The aims of this study were to analyze factors for predicting cardiac death and to create a prediction model incorporating MIBG imaging based on the Japanese multicenter pooled database.

## Materials and methods

### Subjects

Individual datasets from six prospective MIBG cohort studies performed in Japan between 1990 and 2009 were combined to make a pooled database of 1,322 CHF patients as previously reported [5, 13–19]. All patients were enrolled in prospective observational studies, following which MIBG was approved for clinical use in Japan. The diagnosis of CHF was established by cardiologists in each hospital using standard Framingham diagnostic criteria. All the original cohort studies conformed with the principles outlined in the Declaration of Helsinki (Br Med J 1964;2:177). All the studies were approved by the ethics committee or institutional review board in each hospital, and informed consent was obtained from the patients.

To create a single pooled database, cardiology specialists and/or nuclear medicine specialists in six hospitals created databases including common clinical parameters and updated the outcome in some institutions. For this study, only patients with known 5-year outcomes were included (933 patients). The demographics of this population are summarized in Table 1. The underlying diseases were dilated cardiomyopathy (42.6 %), ischemic heart disease (26.3 %), and miscellaneous causes of heart failure (31.1 %) including arrhythmia, hypertrophic cardiomyopathy, secondary cardiomyopathies (diabetes, hypertension, collagen diseases, etc.), valvular heart diseases, myocarditis and cardiac sarcoidosis. Eleven patients (1.2 %) had implantable cardioverter-defibrillators (ICD) and 7 (0.8 %) had cardiac resynchronization therapy (CRT).

**Table 1:** Demographics of the 933 patients

Characteristic	Value
Age (years), mean ± SD	60.8 ± 13.5
Male gender (%)	71 .0
NYHA functional class (%)	
I	23.7
II	42.0
III	27.6
IV	6.8
LVEF (%), mean ± SD	35.7 ± 13.1
^123^I-MIBG HMR, mean ± SD	
Early	1.86 ± 0.39
Late	1.71 ± 0.33
Time of follow-up to event (years), mean ± SD	7.7 ± 4.3
BNP^a^ (pg/ml), mean ± SD	405 ± 451
Ischemic heart disease (%)	26.3 %
Dilated cardiomyopathy (%)	42.6 %
Complications (%)	
Diabetes	23.2
Dyslipidemia	26.3
Hypertension	28.4
Ventricular tachycardia	29.9
Medications (%)	
Angiotensin converting enzyme inhibitor	41.1
Angiotensin receptor blockers	34.7
Beta blocker	57.4
Loop diuretics	72.4
Aldosterone antagonist	48.7

^a^299 patients

### ^123^I-MIBG study

Anterior planar images using scinticameras equipped with low-energy type collimators were obtained at 15 – 30 min (early phase) and 3 – 4 h (late phase) after injection of 111 MBq ^123^I-MIBG (Fujifilm RI Pharma Co. Ltd., Tokyo, Japan) in all institutions. To calculate a heart-to-mediastinum ratio (HMR), a whole-heart region and a mediastinal rectangular region in the upper mediastinum were drawn. Although both early and late HMRs were calculated in each institution, we used the late HMR for creating the prediction model, since late HMR showed better diagnostic accuracy than early HMR [13]. The mean late HMR was 1.71 ± 0.33.

### Left ventricular function

Left ventricular ejection fraction (LVEF) was determined using a gated blood-pool study (317 patients, 34 %), two-dimensional echocardiography (498 patients, 54 %), gated SPECT (98 patients, 11 %) and either a gated blood-pool study or echocardiography (18 patients, 2 %). The mean LVEF was 35.7 ± 13.1 % (Table 1).

### Outcome

Five-year cardiac death, including heart failure death, sudden cardiac death (witnessed cardiac arrest and death within 1 h of onset of acute symptoms or unexpected death in patients known to have been well within the previous 24 h), and fatal acute myocardial infarction, was determined by the original study investigators. While clinical outcomes were confirmed from patient medical records or telephone interview in each institution, only definitely confirmed cardiac death was considered as an event. Noncardiac death was dealt with as censored at the time of death.

### Statistical analysis

All the clinical information and MIBG results were sent to a central institution (Kanazawa University), and were independently analyzed. Data are expressed as means ± standard deviation (SD). Contingency table analysis was examined using likelihood ratio and Pearson statistics.

Variables included in the multivariate analyses were age, gender, early HMR, late HMR, MIBG washout rate, NYHA functional class, LVEF, history of diabetes, hypertension, dyslipidemia, atrial fibrillation and sustained ventricular tachycardia, and medications (including beta blockers, diuretics, angiotensin-converting enzyme inhibitors, angiotensin receptor blockers and aldosterone antagonists). B-type natriuretic peptide (BNP) was not used in the final analysis because of the limited number of patients available for analysis (299 patients). Variables derived from electrocardiography were not included in the assembled dataset.

Multivariate Cox proportional hazard analysis was performed. Logistic regression analysis was used for single HMR and multiple variables, and receiver operating characteristic (ROC) curves and areas under the curves (AUC) were calculated. To create a prediction model, logistic models with single or multiple variables were used to calculate the cardiac mortality. Inverse prediction of HMR was also performed based on the prediction models to evaluate the threshold for low risk of mortality. Net reclassification improvement (NRI) analysis was based on the prediction models with and without HMR. The 5-year risk levels were defined as low (<5 %), intermediate (5 – 25 %) and high (>25 %). The threshold of 5 % corresponded to an accepted low-risk threshold of 1 % mortality per year. The threshold for high-risk (25 %; 5 % per year) was chosen to exceed the approximately 4 % per year cardiac death rate in the total population. A *p* value <0.05 was considered significant. Statistical analysis was performed using JMP 10.0.2 software (SAS Institute Inc., Cary, NC), and mathematical calculation and software for creating models were based on Mathematica 9 (Wolfram Research Inc., Champaign, IL).

## Results

### Five-year cardiac death

**Fig. 1:**
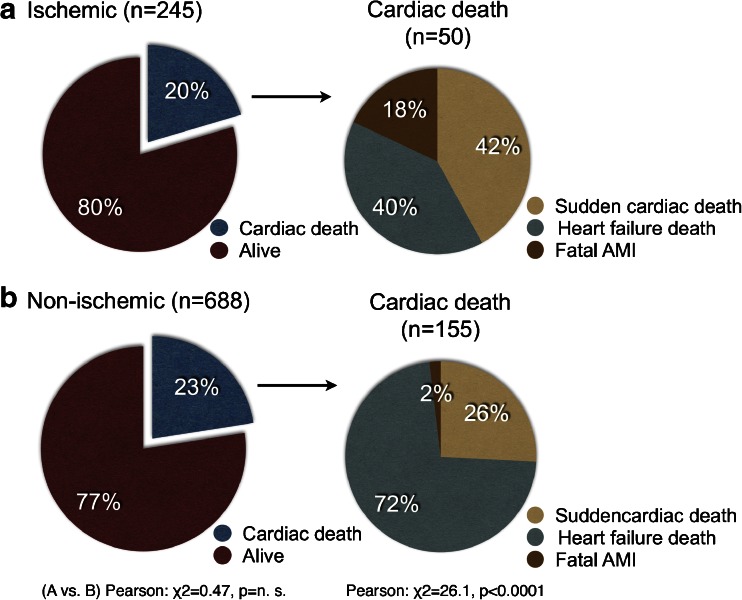
Cardiac death in the ischemic (**a**) and nonischemic (**b**) groups. The cause of death is further classified into sudden cardiac death, heart failure death and fatal acute myocardial infarction (*AMI*). A significant difference was observed in the breakdown of the causes of cardiac death between the ischemic and nonischemic groups (Pearson *χ* ^2^ = 26.1, *p* < 0.0001)

A total of 205 patients (22.0 %) died of a cardiac event, including 132 (14.1 %) with heart failure death, 61 (6.5 %) with sudden cardiac death and 12 (1.3 %) with fatal acute myocardial infarction. Five-year cardiac death rates for NYHA classes I to IV were 20 of 221 (9.1 %), 53 of 391 (13.6 %), 100 of 257 (38.9 %) and 32 of 63 (50.8 %), respectively (*χ* ^2^ = 111.3, *p* < 0.0001, Pearson's analysis). For the following analysis, NYHA functional class was classified into two groups: NYHA classes I/II and III/IV (cardiac death 11.9 % and 41.3 %, respectively; *χ* ^2^ = 105.3, *p* < 0.0001, Pearson's analysis). Five-year death rates in patients with and without ischemic heart disease were 50 of 245 (20.4 %) and 155 of 688 (22.5 %), respectively (not significantly different). However, causes of cardiac death differed between the ischemic and nonischemic patients. In the former group, only 40 % of deaths were due to progressive heart failure, compared with 72 % in the latter group (*p* < 0.0001; Fig. 1). Conversely, the incidence of sudden cardiac death and fatal acute myocardial infarction was higher in the ischemic group than in the nonischemic group.

### Univariate logistic regression analysis with HMR

A univariate logistic model using HMR was applied to patient groups with NYHA classes I/II and III/IV, and ROC analysis was performed. In patients with NYHA functional classes I/II, parameter estimates of HMR showed *χ* ^2^ = 36.6 and a unit odds ratio (OR) of 0.063; the ROC AUC was 0.717 (*p* < 0.0001). In patients with NYHA classes III/IV, parameter estimates of HMR showed *χ* ^2^ = 11.6 and a unit OR of 0.251; the ROC AUC was 0.609 (*p* = 0.0007).

### Proportional hazard analysis

Cox proportional hazard analysis was performed using the variables listed in Table 1. In the multivariate proportional hazard analysis NYHA functional class, late HMR, age, gender and LVEF were significant variables for predicting 5-year cardiac mortality (Table 2).

**Table 2:** Cox proportional hazard analysis for 5-year cardiac death

Variable	Hazard ratio	Lower 95 %	Upper 95 %	Reciprocal of hazard ratio	*p* value
Age	1.024	1.013	1.035	0.977	<0.0001
NYHA I/II vs. III/IV	2.574	1.980	3.359	0.388	<0.0001
Late HMR	0.246	0.156	0.386	4.064	<0.0001
Male gender	1.524	1.146	2.054	0.656	0.0035
LVEF	0.982	0.970	0.993	1.019	0.0035

### Five-year logistic regression analysis

In the multivariate logistic regression analysis without HMR significant variables were NYHA functional class (OR 4.3, *χ* ^2^ = 66.7, *p* < 0.0001), age (OR 1.02, *χ* ^2^ = 11.6, *p* = 0.0007), LVEF (OR 0.98, *χ* ^2^ = 11.3, *p* = 0.0008) and male gender (OR 1.8, *χ* ^2^ = 7.9, *p* = 0.0049). The ROC AUC was 0.749 (95 % confidence interval, CI, 0.709 – 0.785). In the logistic regression analysis with HMR significant variables were NYHA functional class (OR 3.7, *χ* ^2^ = 51.4, *p* < 0.0001), HMR (OR 0.15, *χ* ^2^ = 31.5, *p* < 0.0001), age (OR 1.03, *χ* ^2^ = 11.9, *p* = 0.0006), male gender (OR 1.7, *χ* ^2^ = 6.5, *p* = 0.011) and LVEF (OR 0.98, *χ* ^2^ = 5.1, *p* = 0.024). The ROC AUC was 0.780 (95 % CI 0.744 – 0.813). Of the two ROC curves, the model including HMR had a significantly larger AUC (*p* = 0.0015).

### Reclassification improvement analysis

The NRI analysis was performed using the four-parameter logistic model created by the combination of NYHA functional class, age, gender and LVEF (model 1) and by the five-parameter model that included late HMR (model 2; Table 3). Of the 205 patients who died of a cardiac event, classification was improved in 23 and made worse in 13 using the five-parameter model. The net gain in reclassification proportion (Table 4) was 4.9 % (*p* = 0.096). Of the 725 patients who did not die of a cardiac event, 38 were reclassified upward and 103 downward, with a net gain in reclassification of −9.0 % (*p* < 0.0001). The NRI in all subjects was 13.8 % (*p* < 0.0001). Thus, model 2 significantly improved the identification of patients at low risk of cardiac death when compared to model 1.

**Table 3:** Reclassification analysis in patients who did and did not die of a cardiac event

NYHA class+age+gender+LVEF	NYHA class+age+gender+LVEF+HMR				
	<5 %	5 – 25 %	>25 %		
Subjects who died					
<5 %	0	2	0		
5 – 25 %	4	52	21		
>25 %	0	9	117		
Subjects who survived					
<5 %	35	7	0		
5 – 25 %	72	405	31		
>25 %	2	29	144

**Table 4:** Net reclassification in patients who did and did not die of a cardiac event

	Improved	Made worse	Same	NRI (%)	*p* value
Subjects who died	23	13	169	4.9	0.096
Subjects who survived	103	38	584	9.0	<0.0001
Entire population				13.8	<0.0001

### Prediction of cardiac death using the logistic model

**Fig. 2:**
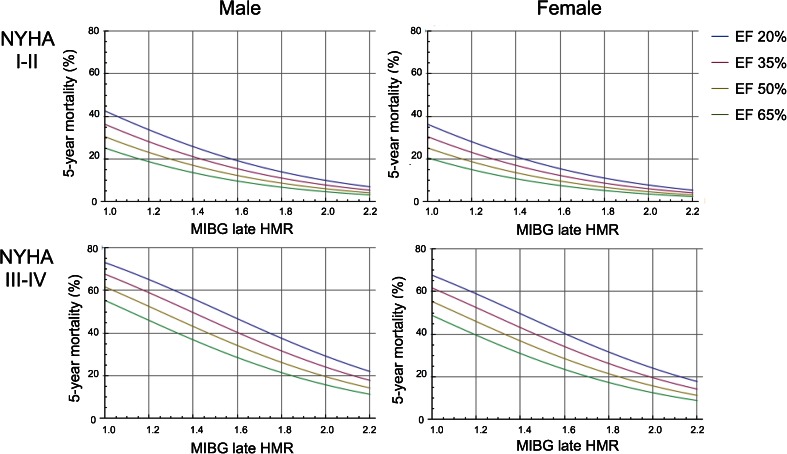
Five-year cardiac mortality based on the five-parameter logistic model. Nomograms for age 60 years are shown for each gender. The nomogram curves are plotted for ejection fractions of 20 %, 35 %, 50 % and 65 % in patients with NYHA classes I/II and III/IV. To use these nomograms, a chart of corresponding age, NYHA class and gender is selected, and the cross-over point of the curves with LVEF and HMR is determined

**Fig. 3:**
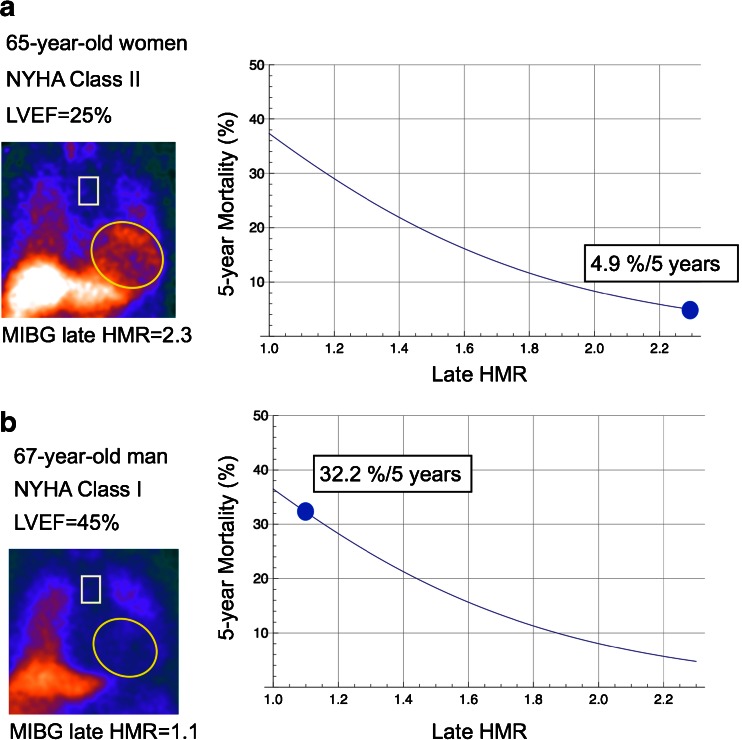
Risk calculation based on five variables. Regions of interest on heart and mediastinum are shown. **a** In a 65-year old female patient with NYHA class II and LVEF 25 %, HMR was 2.3, and the mortality risk could be calculated as 4.9 %/5 years. **b** A 67-year old male patient with NYHA class I and EF 45 % showed HMR of 1.1, and calculated mortality risk was 32 %/5 years. Each prediction curve is drawn as a function of HMR based on the multiple logistic models of 5 parameters. During the follow-up, the first patient was alive for 5 years, and the second patient died from pump failure in 2.6 years after MIBG study

Based on the 5-year logistic regression analysis, mortality rates were estimated using the formula with the five variables (model 2):

$$ \begin{array}{l}\mathrm{f}\left(\mathrm{NYHA}\ \mathrm{f}\mathrm{unctional}\ \mathrm{class},\mathrm{age},\mathrm{gender},\mathrm{HMR},\mathrm{LVEF}\right)=\hfill \\ {}1/\left(1+\mathrm{Exp}\left[-\left({\mathrm{b}}_{\mathrm{intercept}}+{\mathrm{b}}_{\mathrm{nyha}}\ast \mathrm{NYHA}\ \mathrm{Class}+{\mathrm{b}}_{\mathrm{age}}*\mathrm{age}+{\mathrm{b}}_{\mathrm{gender}}*\mathrm{gender}+{\mathrm{b}}_{\mathrm{ef}}*\mathrm{EF}\left(\%\right)+\right.\right.\right.\hfill \\ {}\left.\left.\left.{\mathrm{b}}_{\mathrm{hmr}}*\mathrm{HMR}\right)\right]\right)*100\hfill \\ {}\left(*\mathrm{asterisk}\ \mathrm{denotes}\ \mathrm{multiplication}\right),\hfill \end{array} $$

where the variables *b*_intercept_, *b*_nyha_, *b*_age_, *b*_gender_, *b*_ef_ and *b*_hmr_ were derived from the parameter estimates of the model and were 0.178, 1.297 (*χ* ^2^ = 51.4, *p* < 0.0001), 0.025 (*χ* ^2^ = 11.9, *p* = 0.001), 0.262 (*χ* ^2^ = 6.5, *p* = 0.011), −0.017 (*χ* ^2^ = 5.1, *p* = 0.024) and −1.882 (*χ* ^2^ = 31.5, *p* < 0.0001) respectively. In this formula, NYHA functional class (class I/II, 0; class III/IV, 1) and gender (female, 0; male, 1) were categorical variables, and age, LVEF and HMR were continuous variables. Representative graphs for 60-year-old patients are shown in Fig. 2. The 5-year mortality rate can also be calculated from the risk calculation formula as shown in Fig. 3.

### Prediction of low-risk for cardiac mortality

**Fig. 4:**
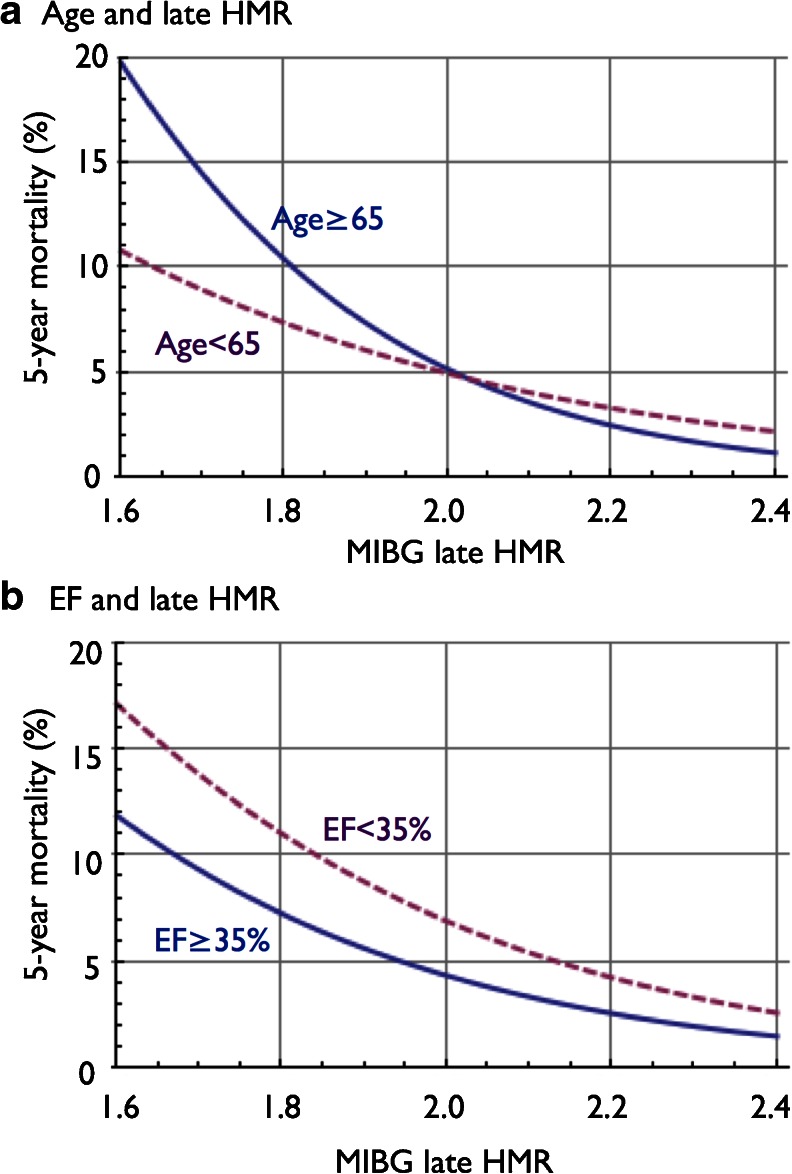
Inverse prediction of 5-year mortality using the logistic model with HMR for patients aged ≥65 years and <65 years with NYHA functional classes I and II

To identify the patients at low risk of cardiac mortality (<5 %/5 years), inverse prediction was performed using the logistic model and a single HMR variable in patients with NYHA classes I/II. Based on the plots of mortality risk versus HMR, patients with NYHA classes III/IV were excluded, since 5 % risk per 5 years was not expected to be associated with HMR in the clinical range. The predicted HMR in patients with a 5 % probability of cardiac death in 5 years was 2.02 (95 % CI 1.88 – 2.27). In patients aged ≥65 years and <65 years, the predicted HMR was 2.01 (95 % CI 1.86 – 2.34) and 2.00 (95 % CI 1.79 – 2.67), respectively (Fig. 4a). In patients with LVEF >35 % and ≤35 %, the predicted HMR was 1.95 (95 % CI 1.79 – 2.32) and 2.14 (95 % CI 1.90 – 2.89), respectively (Fig. 4b).

## Discussion

In this study MIBG HMR was a strong predictor of 5-year cardiac mortality in CHF patients. The ROC and NRI analyses showed additional value of MIBG over conventional clinical parameters. In the logistic regression models the variables age, gender, NYHA functional class, LVEF and MIBG HMR were significant predictors of cardiac death. The contribution of MIBG was particularly significant in reclassifying patients as lower risk. In CHF patients with NYHA class I/II the HMR was 2.0 (associated with a cardiac death rate of 5 % in 5 years).

Accepted prognostic markers for CHF patients include NYHA functional class, LVEF, BNP levels, and electrocardiographic indices such as QRS duration [10, 20]. The present analysis using pooled data from several prospective cohort studies showed that assessment of cardiac sympathetic innervation using MIBG had independent and additive value for estimating the risk of a fatal cardiac event in a wide range of CHF patients over a long follow-up period. As patient-level data were used in the analyses, the results do not have many of the limitations of those from single-center studies or standard meta-analyses based on published data. The significant number of patients and the long follow-up also contribute to the quality of the combined results. This was made possible by the fact that MIBG has been used in the Japanese healthcare system and reimbursed by health insurers since 1992, allowing accumulation of ample experience with the use of MIBG imaging in CHF [9, 13]. This background in Japan thus enabled the creation of prediction models over the long term in CHF patients in the present study.

Based on the proportional hazard and logistic regression analyses, five parameters (NYHA functional class, age, gender, LVEF and HMR) were selected for the prediction model. Of these parameters, dichotomous NYHA functional class showed the highest OR and *χ* ^2^ value, which might be anticipated given the substantially poorer prognosis in CHF patients with NYHA classes III and IV. Inclusion of HMR reduced the prognostic contribution of LVEF to the model, consistent with the results for all-cause death in the full population, where MIBG showed better discrimination between high-risk and low-risk CHF patients than LVEF [13]. This result may correspond to previous findings that there is a non-negligible fraction of high-risk heart failure patients with preserved LVEF and that cardiac MIBG assessment can identify these high-risk patients with preserved LVEF [21].

Clinically useful diagnostic tools which may aid therapeutic decisions have been developed, and several have examined the additive value of MIBG imaging. The Seattle Heart Failure Model is one of the validated prediction models for estimating mortality risk [10, 20]. This model, which includes commonly obtained clinical data such as age, gender, NYHA functional class, medication, and laboratory values including sodium, hemoglobin and cholesterol, has been shown to be improved by addition of HMR [11]. In one small study, a multivariate Cox analysis revealed that the MIBG washout rate and Seattle Heart Failure Model score were independent predictors of cardiac death. A subanalysis of patients in the ADMIRE-HF trial also showed that the addition of MIBG imaging to the Seattle Heart Failure Model improved risk stratification [12]. Thus, further study is warranted to examine the performance of the present proposed model in conjunction with a validated clinical model, as the performance of a combined assessment might surpass that of either model individually.

In contrast to the Seattle Heart Failure Model, which included more than 15 variables, a simpler model using a smaller number of readily available clinical variables and MIBG might be more convenient for clinicians involved in the management of CHF patients. An advantage of our five-variable model is that LVEF and MIBG parameters are available from current quantitative software and can be incorporated into a final computer output.

Although data from follow-up of nearly 15 years were available in some patients in the original database, the rationale for selecting the 5-year mortality analysis was that this time frame is relevant to the use of most current therapeutic approaches in CHF patients. Risk estimation over 5 years represented a compromise in terms of practical clinical significance, since outcomes during longer follow-up can be affected by many factors, including changes in medication, comorbidities and progression of disease. This type of prediction model has been presented using myocardial perfusion imaging but not previously with MIBG studies, and could facilitate more effective use of the test to understand the risk of future events [22, 23]. The present model and nomograms could provide more precise risk stratification and thereby contribute to appropriate selection of therapeutic strategies in CHF patients.

Estimation of mortality risk in patients with CHF could provide a more rational and cost-effective basis for decisions on specific medications and cardiac devices. In particular, identifying low-risk patients would have immediate practical value. For example, in patients with a prior myocardial infarction who might be at high risk of ventricular tachyarrhythmia, the use of prophylactic therapy with an ICD to improve survival [24] might be less desirable if a low long-term mortality risk is demonstrated. In this study, patients with NYHA class I and II and HMR >2.0 had a low risk of cardiac mortality (<5 %/5 years), suggesting primary prevention ICDs might not be necessary in such individuals. Instead, optimal medical therapy might be more appropriate in this population. On the other hand, higher risk patients could be treated using more aggressive medical treatment with ICD and a CRT-defibrillator as judged appropriate. Our previous studies have suggested the efficacy of cardiac MIBG imaging for appropriate ICD indications or for predicting the need for ICD use [25, 26]. The present risk calculations could thus be used in conjunction with developing therapeutic strategies.

As noted previously, the present study showed that the HMR as more useful for reclassifying downward into low-risk groups, opposite to the finding in the ADMIRE-HF subanalysis using the Seattle Heart Failure Model. One possible reason for this difference is the difference in patient populations, with the present analyses including patients with NYHA classes I to IV with average LVEF of 36 %, while the ADMIRE-HF study included only those with NYHA classes II and III with average LVEF of 27 %. The mean HMRs in the two studies were 1.71 and 1.44, respectively, suggesting worse functional status in the ADMIRE-HF study. However, this difference could merely reflect technical differences between imaging equipment in Japan versus gamma cameras in the US and Europe. Another potential explanation is the substantially higher prevalence of patients with non-ischemic HF in the Japanese patients (74 %) versus the ADMIRE-HF patients (34 %) and the lower overall mortality in the Japanese HF patients. However, these observations suggest that MIBG imaging might be more effective in reclassifying upward to a higher risk cohort (such as ADMIRE-HF) and downward to a lower risk population such as that examined in this study.

### Limitations

Since many patients imaged in the 1990s were included in the present study, BNP was not available for all patients, and it could not be included in the model. However, while single BNP measurements often depend on noncardiac factors such as patient condition and medical treatment, HMR using cardiac MIBG activity is relatively stable and may be more appropriate as a baseline assessment for long-term risk. The present prediction model reflects the derivation cohort, and when applied to other CHF populations, the optimal thresholds of HMR may differ. However, in recent reviews and multicenter studies, the HMR thresholds for low-risk patients have been between 1.6 and 1.8, similar to the findings presented here [7–9]. HMR can also vary depending on the types of camera–collimator combinations used, and although it is possible to normalize these results using suitable calibration techniques [27], such standardization was not possible in the present study. Unlike prospective studies in which complete laboratory and ECG data are available for every patient, such data were not available in this study, potentially limiting the scope of the derived model. Validation studies using this MIBG model will be important for advancing its use in clinical patients. There were a greater number of patients with nonischemic heart disease in this Japanese cohort compared with American/European cohorts in other studies of CHF patients [7, 28]. The present study, however, showed comparable cardiac mortality in ischemic and nonischemic CHF groups. Therefore, this prediction model should be valid for both groups, although the ischemic group showed a higher incidence of fatal acute myocardial infarction and sudden cardiac death, whereas the nonischemic group showed a higher incidence of pump failure death.

The rates of use of standard CHF medications such as beta blockers, angiotensin-converting enzyme inhibitors and angiotensin receptor blockers and aldosterone inhibitors were lower (30 – 70 %) than in contemporary CHF studies, but these variables were nevertheless not significant in the multivariate analysis. Considering that the earliest patients in the study were examined prior to routine use of many of these medications in CHF patients and that multiple medication changes might have occurred during long-term follow-up, the relevance of this observation is uncertain. Information on other cardiac medications, such as diuretics and those used to treat arrhythmias, such as amiodarone, was also not included in the database. Of note, the associated conditions of diabetes, dyslipidemia and hypertension were not predictors of cardiac death in the study population.

### Conclusion

The present study confirmed the long-term prognostic value of MIBG imaging in CHF patients and supported development of a potentially useful five-variable prediction model for cardiac death. The prediction model can be used as the basis for graphical nomograms or computer applications to assist clinicians in stratifying risk in their CHF patients. With further validation, these methods could contribute to improvements in the quality and cost-effectiveness of CHF patient care.

## Electronic supplementary material

Below is the link to the electronic supplementary material.

ESM 1 A supplemental figure of all age groups attached for on-line (adjunct to Figure 2). Five-year cardiac mortality based on the logistic model of five variables. Nomograms for ages of 50, 60, 70 and 80 years are shown for men and women. The nomogram curves are plotted for EF of 20 %, 35 %, 50 % and 65 % in subjects with NYHA classes I-II and III-IV. (PDF 714 kb)
