# Supplementary material for: A prediction model for 5-year cardiac mortality in patients with chronic heart failure using 123I-metaiodobenzylguanidine imaging
Source: Eur J Nucl Med Mol Imaging. 2014 Mar 25;41(9):1673–82. doi: 10.1007/s00259-014-2759-x (PMC4122818; doi:10.1007/s00259-014-2759-x)
Supplement: Supplementary file 1 — A supplemental figure of all age groups attached for on-line (adjunct to Figure 2). Five-year cardiac mortality based on the logistic model of five variables. Nomograms for ages of 50, 60, 70 and 80 years are shown for men and women. The nomogram curves are plotted for EF of 20 %, 35 %, 50 % and 65 % in subjects with NYHA classes I-II and III-IV. (PDF 714 kb) [file 259_2014_2759_MOESM1_ESM.pdf]

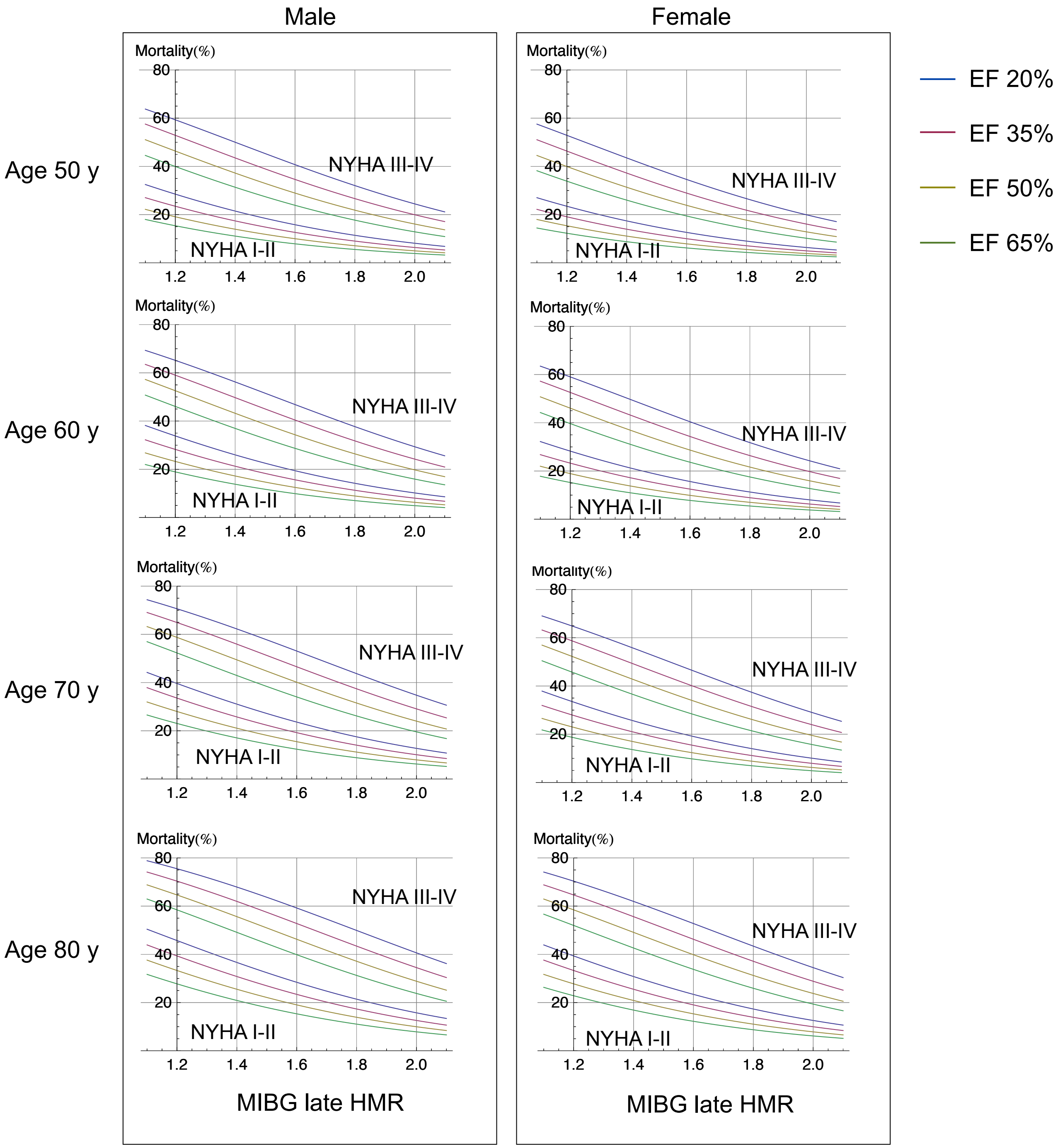

Five-year cardiac mortality based on the logistic model of 5 variables. Nomograms for ages of 50, 60, 70 and 80 years are shown for male and females. The nomogram curves are plotted for EF of 20%, 35%, 50% and 65% in subjects with NYHA classes I-II and III-IV.
